# The Future of Endovascular Therapy for Intracranial Atherosclerotic Disease

**DOI:** 10.1161/SVIN.124.001053

**Published:** 2024-04-14

**Authors:** David S. Liebeskind, Muhammad Bilal Tariq, Naoki Kaneko, Jason D. Hinman

**Affiliations:** ^1^ UCLA Comprehensive Stroke Center, Department of Neurology University of California Los Angeles CA; ^2^ Division of Interventional Neuroradiology, Department of Radiological Sciences University of California Los Angeles CA

**Keywords:** arteries, Editorials, intracranial atherosclerosis, shear stress, stroke

Endovascular therapy (EVT) is a promising strategy for intracranial atherosclerotic disease (ICAD). After SAMMPRIS (Stenting and Aggressive Medical Management for Preventing Recurrent Stroke in Intracranial Stenosis), a landmark trial of EVT in ICAD demonstrated worse outcomes, the US Food and Drug Administration revised indications of the Wingspan stent system to more restrictive criteria.^1^, ^2^ Although on‐label use studies showed significantly reduced risk of periprocedural strokes,^3^ the restrictive labeling means that a substantial number of patients may never qualify for this treatment, yet rates of recurrent stroke remain high in real‐world data and the underlying atherogenic hemodynamic irregularities (ie, focal stenoses) remain uncorrected. EVT with stenting offers the unique possibility to “fix” the focal lesion. At the same time, EVT with stenting, angioplasty, or a combination has revealed potential in studies of acute stroke patients with large vessel occlusion (LVO) due to underlying ICAD (ICAD‐LVO) and prospective studies of “rescue stenting” are ongoing.^4^

How do we *prove* such benefit? To offset potential risks associated with stenting and balloon angioplasty, therapeutic considerations include patient‐specific clinical parameters and their imaging features to identify those *most* at risk of having another event. If we can identify these patients, find which precision medicine theranostic strategy works best for *individual* patients, and *choose* the right end points, benefit can be proven. Beyond individual cases, the future of EVT in ICAD will depend on a practical and rational regulatory strategy, including efficient trial design.

## Use of EVT in Patients with ICAD

When considering patients for EVT, it is essential to first recognize the specific treatment paradigm. Patients presenting with acute LVO and acute ischemic stroke differ from patients presenting with stroke symptoms due to progressive ICAD. Yet whether they present with an LVO or with prior ischemic infarct or evidence of hypoperfusion, the treatment remains the same – reestablish downstream flow and reduce poststenotic proatherogenic and thrombotic stimuli to avert future ischemia.

## Rescue Stenting

Given the acuity and disability associated with LVO, rapid diagnosis and treatment are crucial. However, understanding the LVO etiology may result in different therapy choices, especially as ICAD‐LVO has a higher rate of reocclusion, necessitating use of rescue angioplasty or stenting. Despite being an area of growing interest, challenges remain in early identification of patients with ICAD‐LVO.

Patient characteristics that may help identify ICAD‐LVO patients include absence of atrial fibrillation, a history of hypertension, diabetes, smoking, National Institutes of Health Stroke Scale score <7, and fluctuating symptoms. Studies have focused on identifying patients with ICAD‐LVO, but limited data are available.

Imaging identification of LVOs typically involves noncontrast computed tomography (CT) or magnetic resonance (MR) imaging to evaluate brain parenchyma with vessel imaging on CT angiography or MR angiography. On parenchymal imaging, smaller infarct volume and borderzone infarction patterns are suggestive of ICAD‐LVO. Automated CT perfusion parameters such as lower core and penumbra volumes and low hypoperfusion intensity ratios may also assist with evaluation of collateral status and serve as a proxy for ICAD‐LVO. CT angiography, MR angiography, or digital subtraction angiography features suggestive of ICAD‐LVO include truncal occlusion, robust collateral status, tapered narrowing proximal to the occlusion, and presence of multifocal ICAD. Although these characteristics suggest presence of underlying ICAD, the diagnosis is usually made after initial mechanical thrombectomy. Advanced techniques including intravascular ultrasound and optical coherence tomography may provide information about underlying plaque composition and have potential to guide patient selection for EVT in ongoing work.

The first‐line treatment for ICAD‐LVO remains mechanical thrombectomy. Although no direct comparator studies on initial device or technique choice in mechanical thrombectomy exist for ICAD, one advantage of using stent‐retrievers is the assistance with establishing the diagnosis of ICAD‐LVO if the stent‐retriever conforms to the shape of underlying plaque. Even after diagnosis of ICAD‐LVO, optimal therapy remains unproven. Mechanical thrombectomy frequently does not achieve successful recanalization and rescue EVT is considered. Multiple observational studies have shown that rescue stenting is safe and effective in patients with failed mechanical thrombectomy and may even be considered in patients with successful reperfusion given the high probability of reocclusion and poor outcomes. There has been interest in the use of angioplasty with stenting as first‐line treatment, skipping mechanical thrombectomy altogether. Balloon‐mounted drug‐eluting stents are frequently used,^5^ but there remains a paucity of data on optimal technique. Cautionary variables for use of intracranial stents include large core infarction and concern for hemorrhagic transformation. The use of intravascular ultrasound and optical coherence tomography may improve technical outcomes.

The technical goal of rescue stenting is to provide persistent flow across the stenotic lesion while decreasing risk of reocclusion. To compare techniques, it is important to assess the rate of successful therapy, quality of reperfusion, and rates of reocclusion. However, the primary goal of any treatment is to avert stroke or associated morbidity and mortality. Even after more extensive reperfusion with mechanical thrombectomy, the underlying ICAD stenotic lesion is usually not treated with high risk of recurrent stroke within days or weeks. Studies should ensure long‐term follow‐up as secondary prevention trials have demonstrated that more than half of recurrent strokes occur beyond 3 months.

## Endovascular Therapy for Secondary Stroke Prevention

For secondary prevention, the qualifying event needs to be well‐established. Future trials should consider restricting enrollment to only patients with imaging evidence of stroke unless features that significantly increase stroke risk after transient ischemic attack can be identified. The timing of stenting is another important consideration as early stenting after recent stroke may have a higher risk of periprocedural stroke or death due to unstable plaque and increased potential for embolization. However, the highest risk of stroke recurrence is within the first week after the initial qualifying event. Future studies need to investigate the optimal timing of stent placement. Patients should be enrolled as close to the qualifying stroke event as possible, with strokes before planned stent placement counted as failure of medical therapy in a control arm.

For most ICAD studies to date, patients were enrolled based solely on degree of luminal stenosis. Although an increasing degree of stenosis does confer increased stroke risk, many other factors influence risk of stroke recurrence, including collateral flow, infarction pattern, lesion location, and hemodynamic status, which have traditionally *not* been considered. Perfusion imaging delay and borderzone infarction patterns implying high risk of early neurological deterioration in ICAD are 2 distinct, yet related biomarkers. Ongoing research will determine whether these 2 biomarkers may be leveraged to prompt EVT to avert early recurrent stroke in ICAD.[Fig svi212900-fig-0001]

Advanced imaging modalities are currently being studied to further characterize plaque composition and hemodynamics across the stenoses to further identify patients at highest risk of recurrent stroke. Computational fluid dynamics (CFD) models constructed using CT angiography, MR angiography, high‐resolution MR imaging, or digital subtraction angiography can quantify translesional pressure ratios, shear strain rate, wall shear stress, and signal intensity ratios, all of which are associated with an increased risk of stroke recurrence.^6^ CFD before EVT for ICAD can guide patient selection and postprocedure normalization of these markers may suggest a decreased risk of stroke recurrence (Figure).^7^ Despite multiple articles and research projects on CFD of ICAD, CFD remains largely a research tool and broad adoption or large‐scale implementation of CFD for ICAD characterization in routine clinical practice has not occurred. The SEPIA (Shear Stress and Endothelial Pathophysiology in Intracranial Atherosclerosis) study is underway to assess poststenotic wall shear stress association with endothelial dysfunction using CFD models of cases from SAMMPRIS to construct detailed anatomical flow models.^8^ Similarly, the FCG (Platelet Expression of FcγRIIa and Arterial Hemodynamics to Predict Recurrent Stroke in Intracranial Atherosclerosis) study is investigating the interaction of antiplatelet therapies with FcγRIIa expression on platelets with CFD poststenotic wall shear stress to predict the risk of stroke recurrence (NCT05518305).^9^ The findings from these studies may make CFD part of standard evaluation for risk of stroke recurrence and help select patients to benefit from EVT. High‐resolution vessel wall imaging can be used to identify plaque features associated with increased rates of recurrence although validation in large studies awaits. The CAPTIVA (Comparison of Anti‐coagulation and Anti‐Platelet Therapies for Intracranial Vascular Atherostenosis) study (NCT05907629) is studying MR imaging biomarkers to see if they can predict patients who fail medical management.^10^ Intravascular ultrasound and optical coherence tomography during angiography have the potential to directly visualize plaque to further assist in risk stratification, guide stent position, or expansion but its invasive nature has limited use in patients with ICAD.

**Figure svi212900-fig-0001:**
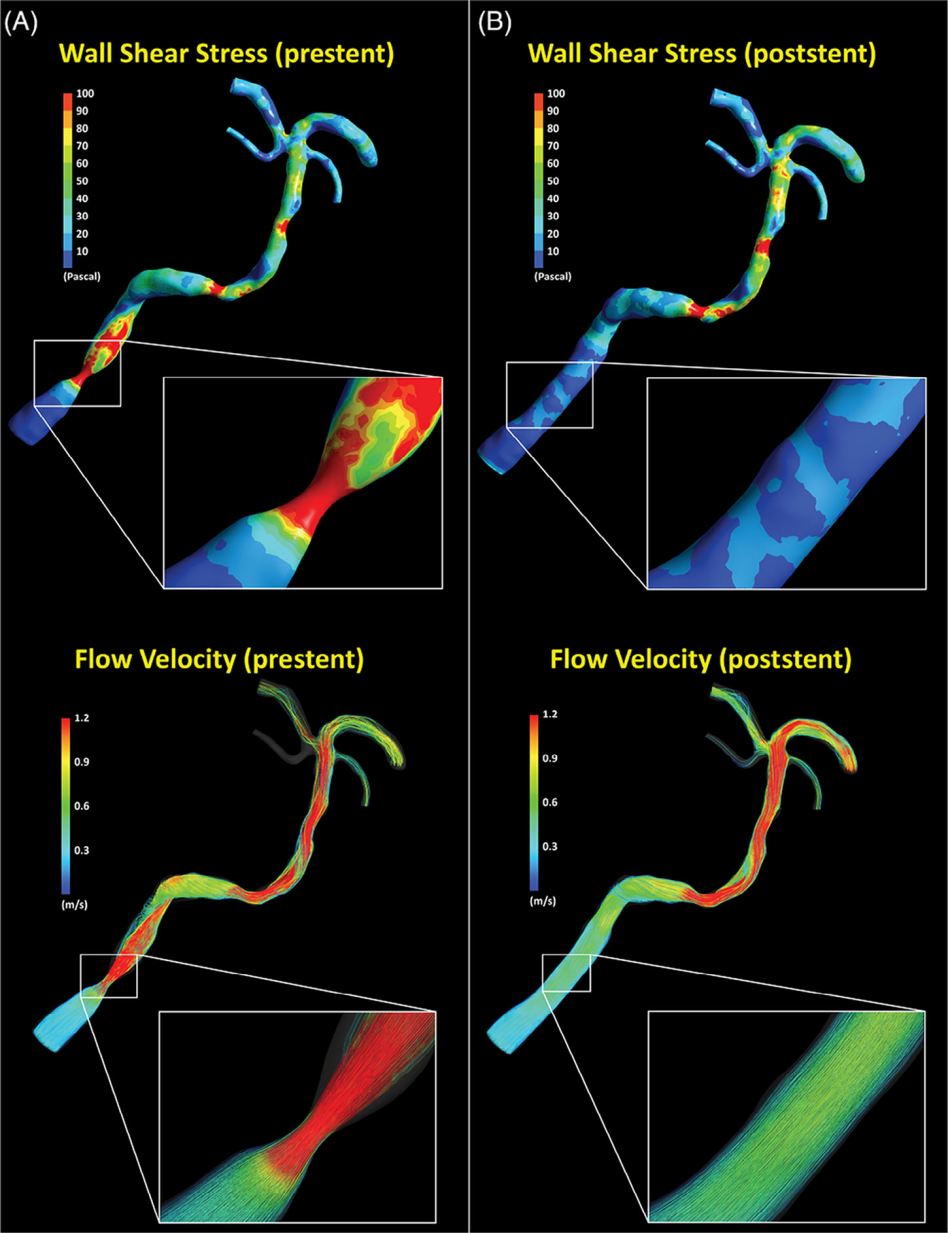
**Wall shear stress changes in intracranial atherosclerosis before and after stenting**. **A**, Areas of low wall shear stress poststenosis (top) and high flow velocity in the stenotic region (bottom). **B**, Demonstrates poststenting changes including normalized wall shear stress in the stented region (top) and reduced flow velocity in the stented region (bottom).

Future studies should both validate features associated with an increased risk of stroke recurrence but also combine multiple features to improve prediction of stroke recurrence in individual patients.

Currently the endovascular treatments commonly used for ICAD include balloon angioplasty, stenting, or a combination of both. The technical goal for any EVT of ICAD is to remodel the deleterious morphology of the focal lesion or at least decrease the level of stenosis and thus reduce proatherogenic effects of the ICAD stenosis.

Balloon angioplasty remains popular due to possibly lower risk, including avoidance of in‐stent thrombosis and the morbidity associated with stent deployment. A more promising version of the therapy, submaximal angioplasty, is currently being studied in the BASIS (Balloon Angioplasty for Symptomatic Intracranial Artery Stenosis) randomized controlled trial (NCT03703635) and has shown a better risk profile against historical controls.^11^ Small studies of patients with ICAD suggest that use of drug‐coated balloons may further improve efficacy of balloon angioplasty by using antiproliferative compounds. One limitation to studying balloon angioplasty as a primary therapy is the need for stenting in case of angioplasty failure, which may introduce bias in analyzing patients treated only with angioplasty. However, a combination of angioplasty with stenting, if angioplasty is not successful, may be optimal.

Although the high rate of ischemic stroke in the stenting arm of prior randomized controlled trials was multifactorial,^1^, ^12^, ^13^ the high rate of in‐stent restenosis contributed to a higher‐than‐expected rate of stroke leading to drug‐eluting stents becoming an attractive alternative. Both sirolimus‐eluting and zotarolimus‐eluting stents have shown low rates of in‐stent restenosis and stroke recurrence.^14^, ^15^ Additional studies to demonstrate efficacy are ongoing.^16^

Despite multiple observational studies, optimal device and technique remains elusive. It is likely that using advanced imaging techniques, we may be able to not only predict patients who may benefit from angioplasty alone compared to stenting but also determine which device may best suit atherosclerotic stenosis.

The success of EVT must prevent recurrent ischemia in the downstream territory. Most observational EVT studies include in‐stent restenosis as a primary outcome, which depending on timing and degree of stenosis, may have limited consequence. The primary outcome should remain recurrent ischemia, including transient ischemic attacks, asymptomatic strokes, and symptomatic strokes along with associated morbidity. As the effect of transient ischemic attack and asymptomatic infarcts on long‐term outcomes is not established, cognitive outcomes should form a vital part of serial assessments. To reduce bias against EVT groups, follow‐up imaging should be standardized between both arms to capture a similar number of asymptomatic infarcts. Another important consideration is duration of follow‐up. Most observational studies report follow‐up to 30‐days, with some including data as far out as 1 year. However, extended follow‐ups should be performed to ensure therapies are effective at the peak of stroke recurrence and restenosis between 6 and 18 months.

## Future Trials of Endovascular Therapy in ICAD

An important part of future endovascular trials will be Food and Drug Administration regulatory pathways for the use of devices in clinical studies. The humanitarian device exemption (HDE) program is a regulatory pathway for disease that affects less than 8000 patients each year and needs to demonstrate safety and probable benefit. This limits HDE use to rare pathologies and as such, the Investigational Device Exemption may be a more appropriate regulatory pathway toward testing a device. Intracranial stents are a significant risk device, requiring Food and Drug Administration approval prior to use. The Food and Drug Administration will usually perform a benefit‐risk assessment using available data from preclinical, early clinical and studies conducted outside the United States and disapprove an Investigational Device Exemption application if the risks do not outweigh benefits. These limitations have resulted in no large‐scale trials on intracranial stenting in the United States since the SAMMPRIS and VISSIT (Vitesse Intracranial Stent Study for Ischemic Stroke Therapy) studies and had led to off‐label use of devices approved for different indications. Multiple clinical trials are ongoing in Asian countries and may guide future device trials in the United States.

For patients with ICAD‐associated acute LVO, equipoise remains in the use of EVT. A future trial would require randomization to endovascular treatment and nontreatment arms. However, designing such a trial is limited by the limitation of acute diagnosis of ICAD in the setting of time‐dependent treatment. We suggest that any such trial should use prethrombectomy imaging in conjunction with DSA images after 1 pass of thrombectomy to ascertain whether ICAD is the most likely etiology for the LVO and then randomize patients to an EVT arm or solely medical management arm. This would provide further information on both the effect on reocclusion and stroke recurrence.

Currently, no randomized clinical trial has shown benefit of intracranial stenting for ICAD. A future trial of secondary therapy of EVT in ICAD would need to incorporate the aspects discussed earlier to maximize success. Patients at the highest risk of stroke recurrence will need to be identified using advanced imaging, whereas patients at highest risk of procedural complications should be excluded. Using data from ongoing and future studies, the optimal technique, whether balloon angioplasty, stenting, or a combination will have to demonstrate benefit in preventing stroke recurrence in the individual patient's clinical and imaging features. The optimal time when the risk of periprocedural strokes outweighs the risk of stroke recurrence needs to be defined. While waiting for planned EVT, these patients should continue on best available medical treatment, which currently includes dual antiplatelets. Several studies have demonstrated that experienced neurointerventionalists have better outcomes.

## Conclusions

EVT remains a promising strategy for patients with ICAD. Although identification of imaging biomarkers associated with an increased risk of stroke has improved, much work remains in identifying patients at highest risk of stroke recurrence. Prospective observational registries and retrospective analysis of prospectively collected data will help. Results from studies using angioplasty with drug‐coated balloons, intracranial stenting with drug‐eluting stents, and rescue stenting in patients with LVO are encouraging and set the stage for next‐generation randomized controlled trials, using improved patient selection criteria, better devices, and more experienced neurointerventionalists.

## Sources of Funding

NIH R01NS112799, R01NS123734, UG3NS130228.

## Disclosures

Liebeskind: Consultant as Imaging Core Lab to Cerenovus, Genentech, Medtronic, Rapid Medical, Stryker. David Liebskind serves on the Editorial Board of S:VIN. Editorial Board Members are not involved in the handling or final disposition of submissions.
